# Efficacy of l-glutamic acid, N,N-diacetic acid to improve the dietary trace mineral bioavailability in broilers

**DOI:** 10.1093/jas/skaa369

**Published:** 2020-11-17

**Authors:** Gavin M Boerboom, Ronald Busink, Coen H Smits, Wouter H Hendriks, Javier Martín-Tereso

**Affiliations:** 1 Trouw Nutrition R&D, Amersfoort, MH, The Netherlands; 2 Animal Nutrition Group, Department of Animal Sciences, Wageningen University, Wageningen, WD, The Netherlands

**Keywords:** broiler, L-glutamic-acid-N-N-diacetic-acid, trace mineral, zinc

## Abstract

Trace minerals are commonly supplemented in the diets of farmed animals in levels exceeding biological requirements, resulting in extensive fecal excretion and environmental losses. Chelation of trace metal supplements with ethylenediaminetetraacetic acid (**EDTA**) can mitigate the effects of dietary antagonists by preserving the solubility of trace minerals. Lack of EDTA biodegradability, however, is of environmental concern. l-Glutamic acid, N,N-diacetic acid (**GLDA**) is a readily biodegradable chelating agent that could be used as a suitable alternative to EDTA. The latter was tested in sequential dose–response experiments in broiler chickens. Study 1 compared the effect of EDTA and GLDA in broilers on supplemental zinc availability at three levels of added zinc (5, 10, and 20 ppm) fed alone or in combination with molar amounts of GLDA or EDTA equivalent to chelate the added zinc, including negative (no supplemental zinc) and positive (80 ppm added zinc) control treatments. Study 2 quantified the effect of GLDA on the availability of native trace mineral feed content in a basal diet containing no supplemental minerals and supplemented with three levels of GLDA (54, 108, and 216 ppm). In study 1, serum and tibia Zn clearly responded to the increasing doses of dietary zinc with a significant response to the presence of EDTA and GLDA (*P* < 0.05). These results are also indicative of the equivalent nutritional properties between GLDA and EDTA. In study 2, zinc levels in serum and tibia were also increased with the addition of GLDA to a basal diet lacking supplemental trace minerals, where serum zinc levels were 60% higher at the 216 ppm inclusion level. Similar to the reported effects of EDTA, these studies demonstrate that dietary GLDA may have enhanced zinc solubility in the gastrointestinal tract and subsequently enhanced availability for absorption, resulting in improved nutritional zinc status in zinc-deficient diets. As such, GLDA can be an effective nutritional tool to reduce supplemental zinc levels in broiler diets, thereby maintaining health and performance while reducing the environmental footprint of food-producing animals.

## Introduction

Trace minerals, in particular trace metals such as zinc (Zn), copper (Cu), manganese (Mn), and iron (Fe), are essential to ensure health and performance in highly productive farm animals. To fulfill the biological requirement for these trace minerals, animals should receive sufficient levels of a bioavailable source (Brugger and Windisch, 2017, 2019; Goff, 2018; Skrypnik and Suliburska, 2018). In commercial poultry diets, it is common to supply trace minerals as inorganic sources (i.e., sulfates and oxides). The most common inorganic sources typically undergo hydrolysis into the metal ion form during digestion, leaving them susceptible to precipitation with dietary antagonists such as phytate, which reduces their nutritional availability. Consequently, nutritionists formulate diets where mineral inclusion is in amounts many fold higher than the quantity retained by the animals, resulting in excessive excretion (Brugger and Windisch, 2015). As such, the fate of dietary trace minerals, particularly Cu and Zn, can be an environmental burden, and improving the bioavailability of trace minerals is an important step toward more sustainable animal food production (Moore et al., 1995; Dozier et al., 2003; Burrell et al., 2004).

Organic trace minerals, in which the mineral links by chelation to organic ligands, such as amino acids or organic acids, are also used in animal nutrition. Organic complexation maintains the solubility of trace minerals within the digestive tract, thereby preserving their bioavailability (Ao et al., 2009; Richards et al., 2010; Star et al., 2012). Although not commonly used in nutritional formulation, strong chelating agents can also increase the bioavailability of trace metals by maintaining the solubility of these elements during the process of digestion (Vohra and Kratzer, 1964, 1968). Strong chelating agents, such as ethylenediaminetetraacetic acid (**EDTA**), are molecules with a high affinity to form strong complexes with trace metals and maintain the stability of the mineral complex in the upper gastrointestinal tract, which minimizes the formation of insoluble molecules (Vohra and Kratzer, 1964, 1968; Whittaker and Vanderveen, 1990). The binding strength of these chelators is many exponents greater than that of small organic ligands such as amino acids or organic acids. Strong chelating agents represent an opportunity to both lower inclusion levels of trace minerals while reducing fecal losses to the environment. EDTA has been proven to enhance the nutritional availability for trace metals (Forbes, 1961; Davis et al., 1962; Vohra and Kratzer, 1964, 1968); however, it may not be a suitable solution to reduce environmental losses because of its limited biodegradability and accumulation in soils and surface waters (Bucheli-Witschel and Egli, 2001). l-Glutamic acid, N,N-diacetic acid (**GLDA**) is a readily biodegradable alternative to EDTA. This molecule could be considered as an environmentally friendlier alternative to EDTA, with a relatively high chelation affinity for relevant trace metal nutrients and with a much lower environmental persistency, with more than 60% being degraded within 28 d (Borowiec et al., 2009; Kołodyńska, 2013; Wu et al., 2015). To date, little information is available on the efficacy of GLDA toward enhancing dietary trace mineral availability in animals (ECHA, 2010). The suitability of this application may be affected by the animal species in question, supplemental levels of the trace elements and of the chelating agent, gastrointestinal conditions, and the chemical affinity of the ligand for the different trace elements and for the other much more abundant metals such as Ca. This manuscript aimed to investigate the effect of GLDA on trace mineral availability in diets high in Ca and phytate, which are relatively well-understood antagonists. Two studies ran simultaneously with different objectives. The first study investigated the GLDA effect on Zn sulfate when added in molar amounts with equal chelation capacity to the added Zn and compared the effect with EDTA, also added in equal chelation capacity to Zn sulfate. The second study investigated the effect of GLDA on trace mineral availability of basal feed minerals (without supplemented Zn, Cu, Fe, and Mn). It was hypothesized that GLDA would improve trace mineral availability.

## Materials and Methods

### Animals

The studies ran simultaneously and were designed and carried out in full compliance with Spanish legislation for the welfare of experimental animals. A total of 1,728, 1-d-old, Ross 308 male broilers (Ross 308, Aviagen, Huntsville, AL, USA) were sourced from a commercial hatchery (SADA, Cazalegas, Toledo, Spain) where birds had been vaccinated against coccidiosis, infectious bronchitis, and Marek’s disease. Upon arrival at the research center (Trouw Nutrition Poultry Research Centre, Casarrubios del Monte, Toledo, Spain), birds were randomly distributed and assigned to 96 pens with 18 animals per pen (1.25 m^2^). The pens were located in two rooms with similar characteristics and pine wood shavings as litter. Pens were blocked by proximity and similarity in three groups of 16 pens per room with treatments randomly assigned within each block. Study 1 consisted of 72 pens, having 6 pens per treatment, with 12 pens assigned to the negative control for better baseline estimation. In total, 24 pens were assigned to study 2 with 6 pens per treatment. All treatments were equally distributed over block and room.

### Diets

In the first 6 d of the trial, all chicks received a standard mineral-adequate starter diet, formulated to fulfill all nutrient requirements (NRC, 1994). On day 7, birds received the experimental diets until day 21 of age. Diets (feeders) and water (nipples) were provided ad libitum.

Treatments consisted of 15 differently formulated diets. The 11 diets used for the first study contained a basal premixture formulated to meet or exceed all nutritional requirements with the exception of Zn (NRC, 1994). Diets included a negative control without added Zn and a positive control with 80 ppm of supplemental Zn. Three levels of supplemental Zn sulfate (5, 10, and 20 ppm of Zn) were fed alone or in combination with molar GLDA or EDTA equivalents to chelate 5, 10, and 20 ppm of Zn (27, 54, and 108 mg/kg feed; Trouw Nutrition, Amersfoort, the Netherlands, 26, 51, and 103 mg/kg feed; Sigma-Aldrich, St Louis, MO, USA) making nine different diets. The four diets of study 2 included a negative control and three incremental levels of GLDA (54, 108, and 216 mg/kg feed). These levels are the molar GLDA equivalents to chelate 10, 20, and 40 ppm of Zn, based on an in vitro assessment in which the amount of soluble Zn was measured after 6-h incubation with a chelator and feed (Trouw Nutrition, unpublished). A basal meal was formulated for these four diets to fulfill or exceed all nutritional requirements, with the exception of Zn, Cu, Mn, and Fe, which were not supplemented (NRC, 1994).

The basal feed for all the diets used in both studies was a combination of corn (15%), wheat (30%), soybean meal (29%), and soy oil (6%). To challenge trace mineral availability, an elevated level of total Ca was applied (9.8 g/kg), as well as 15% rice bran inclusion, which increased phytic acid level to 11.3 g/kg. In order to reduce endogenous phytase activity from the feedstuff, the basal meal was pelleted at an elevated temperature (75 °C; Brugger et al., 2014). Representative samples of the diets were taken after production to determine moisture (EC regulation 152/2009, appendix III A), ash, ether extract (EC regulation 152/2009, appendix III H method A), starch, fiber fractions (ISO 6865:2000), and crude protein content (ISO 16634-1:2008). Calcium, Zn, Cu, Mn, and Fe content was analyzed in duplicate using inductively coupled plasma mass spectrometry (**ICP-MS**) after calcination and HCl extraction according to the method NEN-EN 15510 (Bikker et al., 2017). GLDA content was analyzed in duplicate by liquid chromatography-mass spectrometry (LC-MS) (Masterlab B.V., Boxmeer, the Netherlands). Phosphorus was analyzed by spectrophotometry (official methods of analysis [AOAC], method 4.8.14). Phytic acid was analyzed by the colorimetric AOAC method number 965.17, based on the reaction of vanadomolybdate on inorganic phosphate produced by the action of 6-phytase on phytic acid-containing substrate (Novo et al., 2018). 

### Measurements

General performance including body weight, body weight gain, feed intake, daily weight gain (**DWG**), and feed conversion ratio were determined between days 7 and 21. At the end of the study (day 21), blood samples were taken from the wing vein of three randomly selected birds from each pen. An aliquot of blood was centrifuged for 30 min, the serum collected, and divided into two aliquots of 1 mL per bird in labeled 2.5 mL cryotubes. Serum and whole blood samples were stored at −20 °C until further analysis. Serum Zn, Cu, Mn, and Fe were analyzed by the Scottish Trace Elements and Micronutrient Reference Laboratory (Glasgow, UK) using ICP-MS (Agilent series 7500ce). The samples were diluted 20-fold in a solution of 2% butanol, 0.1% EDTA, 0.2% triammonium citrate, 0.1% triton-X-100, and 2% ammonia, with 50 ug/L germanium as internal standard. Hemoglobin in fresh blood was measured with a HemoCue Hb 201+ (HemoCue Diagnostics BV, Waalre, the Netherlands). After blood collection, the birds were anesthetized by intramuscular injection of a solution made of 50 mL sedamun and 30 mL ketamine (1 mL/kg body weight) and 20 min later euthanized by an intravenous injection of T61 (an aqueous solution containing 200 mg embutramide, 50 mg mebezonium iodide, and 5 mg tetracaine hydrochloride per milliliter). Left and right tibias were dissected out and stored at 4 °C until further processing. Tibias were cleaned from soft tissue after boiling in water and analyzed for Zn and Mn content at the Ainia Centro Tecnológico (Paterna, Spain) using microwave digestion, followed by inductively coupled plasma atomic emission spectrometer analysis (Horiba Jobin Yvon, Ultima model). Tibia Zn and Mn values were then pooled by pen.

### Statistical analysis

#### Study 1

Data were analyzed using SAS Studio (SAS Institute Inc., Cary, NC). Performance data, serum Zn and total, and concentration of Zn in tibias were analyzed using the MIXED procedure with diet as a fixed factor and block as a random effect. Significantly different means were identified with a Tukey test (*P* < 0.05). The linear and quadratic effects of sulfate, GLDA, and EDTA were also determined using the MIXED procedure. Regression analysis on serum and tibia Zn response was performed using the NLMIXED procedure. Since Zn absorption is primarily a saturable, carrier-mediated process, it is nonlinear, and using nonlinear regression over data transformation and linear regression is preferred (Miller et al., 2007). The model used for the analysis of Zn availability using EDTA or GLDA was selected based on the best fit and biological meaning (Archontoulis and Miguez, 2015). Parameters used for determining best fit were the Akaike information criterion, root mean squared error, and concordance correlation coefficient. The negative control treatment containing no chelator and no added Zn was used in all three lines as the starting point and the high Zn treatment was used in all three lines to define an assumed homeostatic plateau. The following model was used:

$$\begin{array}{l} Y=Asymptote*\;exp(-\exp\left(-\left(kSul+kEDTA+kGLDA\right)*\left(Zn\;dose-T\right)\right) \end{array}$$

in which:

Y = response parameter, serum and tibia Zn content,

Asymptote = asymptote, representing the maximum response in the Y variable,

k = rate parameter determining the steepness of the curve,

T = inflection point at which the response rate is maximized,

Sul = factor representing only sulfate inclusion (0,1),

GLDA = factor representing dietary GLDA inclusion on top of sulfate (0,1),

EDTA = factor representing dietary EDTA inclusion on top of sulfate (0,1), and

Zn dose = the amount of Zn sulfate added

Significance (*P* < 0.05) between the three fitted models (sulfate, EDTA, and GLDA) was determined using NLMixed, and the optimal model was used to determine the Zn supplementation required to reach a response of 95% of the asymptotic value for serum Zn and Zn concentration in tibia ash. This value was considered as a criterion for estimating the bioavailability of the Zn in the diet (Huang et al., 2013).

#### Study 2

Data were analyzed using SAS Studio (SAS Institute Inc., Cary, NC). Performance parameters, serum minerals, hemoglobin, and bone mineral concentrations were analyzed using the MIXED procedure, with GLDA inclusion level as a fixed factor and block as a random effect. Significant different GLDA means were identified with a Tukey test (*P* < 0.05). The linear and quadratic effects of GLDA were also determined using the MIXED procedure. Animal performance data also included initial weight as a covariate in cases where this effect was significant. Zinc levels in serum and tibia were subsequently analyzed using the NLMIXED procedure using the following model:

$$\begin{array}{l} Y=Asymptote*\;exp(-\exp\left(-k*\left(GLDA\;dose-T\right)\right) \end{array}$$

Y = response parameter, serum and tibia Zn content,

Asymptote = asymptote, representing the maximum response in the Y variable,

k = rate parameter determining the steepness of the curve,

T = inflection point at which the response rate is maximized, and

GLDA dose = the amount of GLDA added

## Results

### Study 1

Analyses of the feed confirmed the high levels of Ca and phytic acid intended by design and the required Zn, EDTA, and GLDA levels (Table 1). The MIXED and NLMIXED procedures, therefore, used the anticipated Zn, GLDA, and EDTA levels as a dose–response continuous variable. No significant differences were detected in DWG and FCR between treatments in study 1 (diets 1 to 11). Significant differences were observed for feed intake between the treatments at the 5 mg/kg supplementation with birds fed the EDTA having a higher intake compared with the birds fed the GLDA. No difference was present at any of the other dosages (Table 2).

**Table 1.** Calculated and chemically analyzed (between brackets) nutrient composition of the starter feed and experimental feed

Nutrient composition, g/kg	Starter feed (0 to 7 d)	Experimental diet (7 to 21 d)
Dry matter	889.7 (881.4)	895.7 (894.0)
Crude protein	220 (206.9)	220.0 (212.2)
Ash	61.8 (54.0)	71.13 (61.1)
Crude fiber	28.3 (26.0)	34.0 (34.5)
Non digestible fiber	107.1 (107.6)	123.2 (127.2)
Acid Detergent fiber	*n.a.^1^* (36.2)	*n.a.* (40.9)
Acid detergent lignin	*n.a.* (7.0)	*n.a.* (10.3)
Ether extract	72.2 (59.0)	100.0 (95.7)
Starch	389.2 (396.0)	334.0 (337.0)
Ca	9.2 (*nd^1^*)	9.2 (9.8)
P	7.5 (*nd*)	10.3 (9.7)
Phytic acid	2.6 (*nd*)	10.6 (11.3)

1n.a. = not analyzed; nd = not determined.

**Table 2.** Least square mean performance values of broilers receiving non-chelator (none) and EDTA- and GLDA-containing diets with increasing levels of Zn from day 7 to 21

Parameter	Chelator	Zn inclusion level, mg/kg					Model		SEM
		0	5	10	20	80	Linear	Quadratic	
Body weight, day 7									
	None	204	205	202	207	202	0.55	0.43	0.7
	EDTA	—	205	206	201	—	0.39	0.25	
	GLDA	—	203	203	208	—	0.32	0.15	
Body weight, day 21									
	None	1,087	1,081	1,074	1,086	1,061	0.95	0.6	3.7
	EDTA	—	1,099	1,098	1,089	—	0.07	0.09	
	GLDA	—	1,066	1,091	1,100	—	0.24	0.08	
DWG, day 7 to 21									
	None	63.0	62.6	62.2	62.9	61.3	0.78	0.90	0.2
	EDTA	—	63.9	63.8	63.4	—	0.13	0.22	
	GLDA	—	61.6	63.4	63.9	—	0.48	0.28	
Daily feed intake, day 7 to 21									
	None	90.9	90.8^ab^	88.7	89.0	88.5	0.02	0.05	0.3
	EDTA	—	91.6^a^	90.8	89.9	—	0.51	0.36	
	GLDA	—	87.3^b^	89.4	90.9	—	0.001	0.001	
Feed conversion rate, day 7 to 21									
	None	1.44	1.45	1.43	1.42	1.45	0.001	<0.0001	0.002
	EDTA	—	1.43	1.42	1.42	—	0.06	0.36	
	GLDA	—	1.42	1.41	1.43	—	<0.0001	0.001	

^a,b^Values with different superscripts within a column are significantly different (*P* < 0.05).

**Figure 1.**
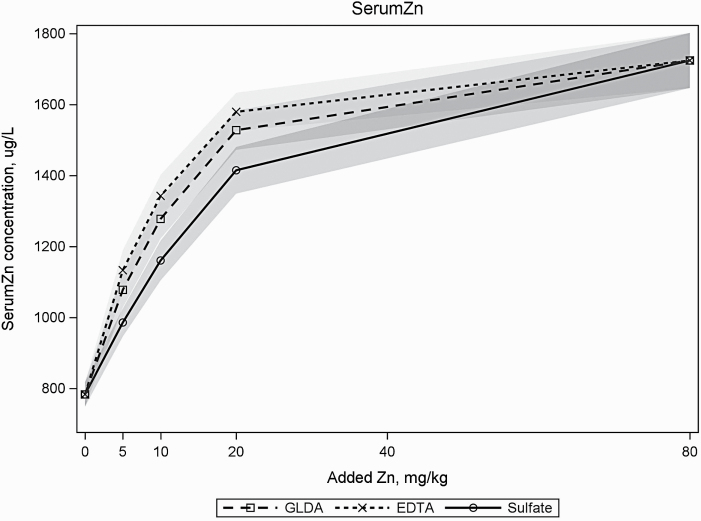
Response of serum Zn levels in broilers when fed dietary Zn supplementation with Zn sulfate, EDTA, and GLDA. Gray area indicates 95% confidence interval.

Serum and tibia Zn content clearly responded to increasing doses of dietary Zn, and this response was strongly significantly affected by the supply of equimolar amounts of EDTA or GLDA in the diets (Table 3). The results of the nonlinear regression analysis showed a clear dose–response effect for Zn on serum Zn, tibia Zn, and total tibia Zn values (Table 4; Figure 1). The tibia and serum Zn data showed a similar response for both EDTA and GLDA. Significant differences between the two chelators and sulfate were observed but not between EDTA and GLDA (Table 4). The estimated dietary Zn level to reach 95% of the model asymptote determined from serum and tibia Zn concentration when EDTA and GLDA were included in the diet were, on average, 69.5% and 68.6% of the estimate when Zn sulfate was used, respectively (Table 4). Also when the total tibia Zn amount was used as a response criterion, the estimated dietary Zn level was 71.0% (EDTA) and 69.6% (GLDA) of the Zn sulfate estimate.

**Table 3.** Least square mean of serum and tibia Zn concentration of broilers receiving non-chelator (none) and EDTA- and GLDA-containing diets with increasing levels of Zn from day 7 to 21

Parameter	Chelator	Zn inclusion level, mg/kg					Model		SEM
		0	5	10	20	80	Linear	Quadratic	
Serum Zn, μg/L									
	None	801	961	1,184	1,382^b^	1,662	<0.01	<0.01	46.5
	EDTA	—	1,110	1,353	1,594^a^	—	<0.01	<0.01	
	GLDA	—	1,007	1,216	1,669^a^	—	<0.01	0.65	
Tibia Zn, mg/kg									
	None	33.8	39.3	42.0^a^	57.0	69.2	<0.01	<0.01	1.69
	EDTA	—	41.2	53.0^b^	63.0	—	<0.01	0.05	
	GLDA	—	41.1	52.2^b^	64.2	—	<0.01	0.19	
Total tibia Zn, μg									
	None	234	269	277^a^	396	481	<0.01	<0.01	14.4
	EDTA	—	278	372^b^	428	—	<0.01	0.06	
	GLDA	—	277	355^b^	433	—	<0.01	0.34	

^a,b^Values with different superscripts within the column are significantly different (*P* < 0.05).

**Table 4.** Parameter of a nonlinear model^1^ describing the response of serum and tibia Zn concentration in broilers to dietary Zn supplementation with Zn sulfate (Sul), EDTA, GLDA, and estimates of Zn requirements (95% of asymptote)

Parameter	Serum Zn, μg/L			Tibia Zn content, mg/kg			Total tibia Zn, μg		
	Estimate	SE	*P*-value	Estimate	SE	*P*-value	Estimate	SE	*P*-value
A	1,730	41.5	<0.0001	72	2	<0.0001	500	18.6	<0.0001
kSul	0.069^a^	0.008	<0.0001	0.055^a^	0.007	<0.0001	0.052^a^	0.009	<0.0001
kEDTA	0.034^b^	0.008	<0.0001	0.022^b^	0.006	<0.0001	0.022^b^	0.007	0.0003
kGLDA	0.021^b^	0.007	0.0032	0.022^b^	0.006	<0.0001	0.019^b^	0.007	0.0002
T	−3.4	0.6	<0.0001	−4.39	0.8	<0.0001	−4.4	1	<0.0001
s2e^2^	12,783	1,845	<0.0001	22.1	3.2	<0.0001	1,796	259	<0.0001
AICC^2^	1,193			583			1,005		
RMSE^2^	113			4.7			274		
CCC^2^	0.94			0.93			0.88		
Supplementary dietary Zn sulfate level (mg/kg) to reach 95% of asymptote									
Sulfate	39.9			50			52.4		
EDTA	25.6			34.4			35.7		
GLDA	29.9			34.2			37.2		

^1^ Y=A×exp(−exp(−(kSul+kEDTA+kGLDA)×(Zndose−T))), where Y = dependent variable (serum Zn, tibia Zn concentration, or total tibia Zn content); A = asymptote; k(Sul, EDTA, GLDA) = rate parameter determining the steepness for sulfate, EDTA, and GLDA, respectively; Zn dose = dietary Zn sulfate supplementation; and T = inflection point at which k is maximized.

^2^AICC, Akaike information criterion; CCC, concordance correlation coefficient; RMSE, root mean squared error; s2e, variance.

^a,b^Values with different superscripts within a row are significantly different (*P* < 0.05).

### Study 2

None of the treatments were significantly different from the control with regard to feed conversion ratio (FCR; Table 5). Furthermore, GLDA supplementation showed significant linear and quadratic effects on DWG (*P* < 0.05) and a trend for daily feed intake (*P* < 0.10).

**Table 5.** Least square mean performance parameters of broilers receiving a basal diet with increasing levels of GLDA from day 7 to 21

Parameter	GLDA inclusion levels, mg/kg				*P*-value		
	0	54	108	216	Linear	Quadratic	SEM
Bodyweight, day 7, g	206	203	203	204	0.35	0.38	1.4
Bodyweight, day 21, g	1,051	1,069	1,080	1,073	0.02	0.05	0.6
DWG, g	60.5	61.8	62.6	62.1	0.02	0.05	0.5
Daily feed intake, g	88.1	90.5	89.8	88.9	0.06	0.06	0.78
Feed conversion ratio, g/g	1.46^ab^	1.47^b^	1.44^a^	1.43^a^	0.35	0.89	0.006

^a,b^Values with different superscripts within a row are significantly different (*P* < 0.05).

**Figure 2.**
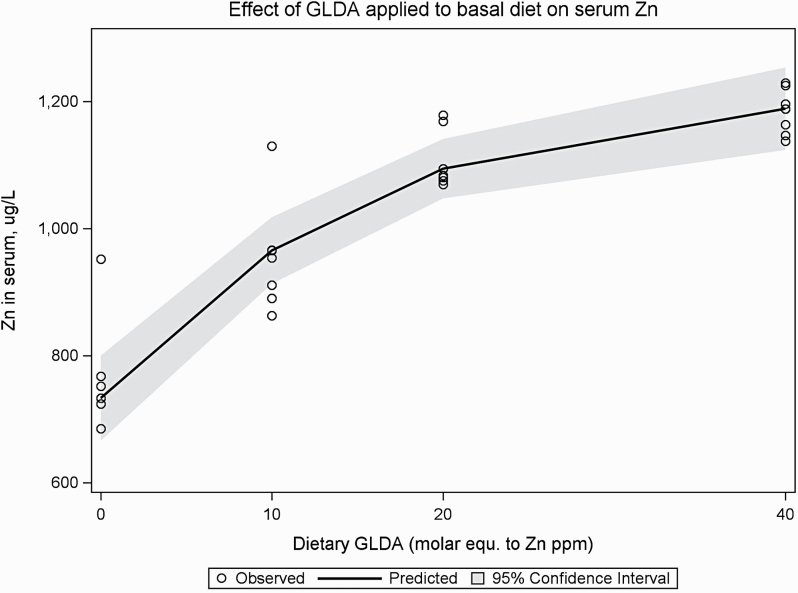
Response of serum Zn levels in broilers when fed increasing dietary GLDA levels in basal diet. Gray area indicates 95% confidence interval.

A linear and quadratic response was observed on serum Zn with a increasing dose of GLDA, while no differences were observed for the other three trace minerals in serum nor for hemoglobin (Table 6). Furthermore, variation in serum Zn appeared to be reduced with increasing GLDA levels (Figure 2). None of the other trace minerals were significantly affected by the addition of GLDA. Bone weight was also unaffected by the dietary treatments. Bone Zn expressed as Zn concentration in the tibia as well as total tibia Zn increased in a similar fashion as serum Zn, increasing with an increasing GLDA dose (Table 6). No differences were observed for Mn levels in the tibia.

**Table 6.** Least square mean mineral concentration in serum and tibia, hemoglobin levels in serum, and tibia weight of broilers receiving a basal diet with increasing levels of GLDA from day 7 to 21

	GLDA inclusion levels, mg/kg				Model		
Parameter	0	54	108	216	Linear	Quadratic	SEM
Serum Zn, μg/L	737^a^	952^b^	1,110^bc^	1,183^c^	<0.0001	<0.0001	39.1
Serum Cu, μg/L	121	121	116	123	ns^1^	ns	4.4
Serum Mn, μg/L	5.9	6.6	7.7	5.9	ns	ns	0.68
Serum Fe, μg/L	2,338	2,379	2,114	1,855	ns	ns	200.9
Hemoglobin, mmol/L	5.80	5.81	5.82	5.84	ns	ns	0.088
Tibia weight, g	6.78	6.74	6.86	6.65	ns	ns	0.179
Bone Zn, mg/kg	31.0^a^	39.5^b^	45.2^bc^	47.2^c^	<0.01	<0.01	1.9
Bone Zn, mg	211^a^	266^b^	310^b^	313^b^	<0.01	<0.01	14
Bone Mn, mg/kg	1.42	1.41	1.51	1.44	ns	ns	0.087
Bone Mn, μg	9.7	9.5	10.4	9.6	ns	ns	0.69

1ns = not significant.

^a,b^Values with different superscripts within a row are significantly different (*P* < 0.05).

The results of the nonlinear regression showed a clear dose–response effect with increasing levels of GLDA in both bone and serum Zn markers (Table 7).

**Table 7.** Estimates of a nonlinear model^1^ describing the response of serum and tibia Zn in broilers to dietary GLDA inclusion in a basal diet

			Tibia Zn			
Parameters	Serum Zn, μg/L		Concentration, mg/kg		Total content, μg	
	Full model	SE	Full model	SE	Full model	SE
A	1,214.4	6.05	48.03	2.08	319.7	4.67
K	0.08	0.003	0.09	0.029	0.098	0.012
T	−8.7	0.28	−9.3	3.07	−8.7	1.07
s2e^2^	6,551	1,891	15.4	4.4	1,070.6	309.1
AICC^2^	289.1		143.8		245.6	
RMSE^2^	80.9		3.92		32.72	
CCC^2^	0.89		0.84		0.76	

^1^ Y=A×exp(−exp(−k×(GLDAdose−T))), where Y = dependent variable (serum Zn, tibia Zn concentration, or total tibia Zn content), A = asymptote, k = rate parameter determining the steepness, GLDA dose = dietary GLDA supplementation, and T = inflection point at which k is maximized.

^2^AICC, Akaike information criterion; CCC, concordance correlation coefficient; RMSE, root mean squared error; s2e, variance.

## Discussion

In the first study, a greater Zn absorption was observed, as indicated by the higher levels of serum and tibia Zn, with increasing doses of Zn in the presence of the two chelating agents tested. These results confirm that both GLDA and EDTA improve the Zn status of broiler chickens. The mode of action, although not studied here, can be expected to be identical to EDTA, namely improving Zn solubility in the gastrointestinal tract allowing for a greater Zn uptake. The second experiment demonstrated that GLDA is able to increase the nutritional availability of native Zn from the basal meal containing no phytase and high phytate. No effects of GLDA on Cu, Mn, or Fe status were observed.

Serum and bone Zn contents are considered a valid indirect indication of Zn absorption (Wedekind and Baker, 1990; Wedekind et al., 1992). Serum Zn can be regarded as a short-term marker for Zn status, whereas bone zinc content can be considered as a responsive criterion for Zn bioavailability in chickens, regardless of low or high dietary trace mineral content (Wedekind et al., 1992; Ammerman et al., 1995; Cao et al., 2002; Huang et al., 2009a, 2009b). It is unclear which expression of bone Zn provides the best assessment of overall Zn status. Dietary Zn influences both total bone Zn and Zn concentration in bone, while total bone Zn can be considered a long-term marker for the Zn status of an animal. However, Zn status affects the overall growth, which may dilute Zn concentration in tissues such as bone. In general, it is important to include the assessment of both serum and bone Zn to address the shortcomings of both (Wedekind et al., 1992). We, therefore, analyzed both in this study.

The incremental response to supplemental Zn was lowest for Zn sulfate when fed alone. The response that was achieved with the addition of EDTA or GLDA can be explained by a chemical inhibition of gastrointestinal precipitation of Zn with other dietary factors, thereby improving Zn solubility. The prevention of Zn binding to phytic acid in poultry by EDTA has been known for some time (Likuski and Forbes, 1964), but data here seem to indicate that GLDA can have a similar effect. The second study also indicated that the availability of the native Zn fraction present in the raw materials is improved by the addition of GLDA, with a greater amount of dietary Zn reaching tissues by incremental doses of GLDA.

Relative differences in tissue Zn are determined not only by Zn supply and availability but also by the Zn status of the animal (Batal et al., 2001; Brugger and Windisch, 2017). Intestinal absorption plays a key role in Zn homeostasis. For this reason, Zn availability is evaluated by rate of response to incremental doses and quantified in relative terms to a reference inorganic source, mainly Zn sulfate, and in this case also EDTA (Edwards and Baker, 1999). Regression analyses estimated that the effect of GLDA on Zn availability is comparable to that of EDTA for all tissues sampled in this trial. This indicates equivalent nutritional properties between these two aminopolycarboxylates. EDTA has been extensively studied for its ability to increase the nutritional availability of trace metals, with emphasis on Fe in humans. These properties are also well demonstrated for Zn and other trace metals in poultry (Davis et al., 1962). This nutritional property is common to many strong chelating agents with stability constants (logK) for Zn between 5 and 20, and the affinity for Zn is quadratically related to increasing dietary Zn concentrations (Vohra and Kratzer, 1964, 1968). The stability constant of the GLDA Zn complex was determined at 10.0 (Kołodyńska, 2011), representing an affinity level that justifies the observed nutritional property described here. EDTA has a stability constant for Zn of 16.5 and considering the GLDA stability constant for Zn it is surprising to see that the two chelators have a similar response in this study (Vohra and Kratzer, 1964). It may be the case that the chelation strength required to reduce precipitation of Zn (by preventing binding to phytate) can already be achieved by using GLDA, giving EDTA no advantage even though its chelation strength is higher. The asymptotes determined in the NLMixed procedure were estimated to be higher than those actually measured in serum and tibia. The measured concentrations, however, were still within the confidence limit of the regression. Having a higher number of birds sampled or more levels tested would have most likely increased this estimation.

Regression analysis for GLDA conducted in the second study indicated that the response lowered near the maximum level of GLDA tested. Typically, a decrease in the response to an increasing availability of Zn is an indication of regulation of absorption as the nutritional requirements are met. The data from study 2 indicate otherwise, as the asymptotes are much lower than in the first study as well as in those found in the literature (Uyanik et al., 2002; Mondal et al., 2010). The observed lower plateau with GLDA may be interpreted as a saturation effect of GLDA in the solubilization of the basal dietary Zn content or it may indicate that there was insufficient Zn present in the basal diet to reach a similar plateau as in the first experiment. Considering that Zn retention of broilers as a fraction of their feed intake is close to 20 ppm (Dewar and Downie, 1984; Batal et al., 2001) and that basal Zn was 32 ppm, it can be speculated that the digestive process was not able to make all dietary Zn available, and hence the asymptote could not be reached.

Incremental doses of GLDA on native Cu showed no effect on serum Cu, indicating that nutritional status was already adequate. Serum levels of Mn also remained unchanged by incremental doses of GLDA. Iron status was studied by serum Fe and blood hemoglobin, and serum Fe was found to be in the range of that described for adequately fed animals at 20 d (Mondal et al., 2010). Therefore, the regulation of absorption may have overshadowed any difference in Fe availability created by GLDA. This conclusion is also supported by the blood hemoglobin data, which were already similar in the negative control diet to that described for healthy broiler chickens at 21 d of age (5.83 ± 0.12 mmol/L; Martinez and Diaz, 1996). Hemoglobin remained unchanged with increasing doses of GLDA. A highly regulated factor such as blood hemoglobin would only respond to an increased dietary availability of Fe in conditions of deficiency. The present data cannot confirm or reject the hypothesis that GLDA has a positive effect on Fe availability as described for EDTA (being similar in effect) in humans (Viteri and Garcia Inbanez, 1978; Viteri et al., 1978; Hurrell et al., 2000) and rats (Whittaker and Vanderveen, 1990). This effect has not been investigated in broiler chickens, most likely because it is difficult to induce Fe deficiency in the animal model (Davis et al., 1962).

Chelation is a promising tool to reduce the fecal output of Zn in broiler chicken production by allowing the safe reduction of Zn inclusion in the feed. The results from the regression analysis indicate that a potential reduction of 15 to 20 mg/kg in dietary Zn supplementation would not compromise the Zn supply of the broilers and the physiological status of the birds with dietary supplementation of EDTA or GLDA. However, because aminopolycarboxylates go unabsorbed through the gastrointestinal tract (Zhu et al., 2006), EDTA would be excreted via the feces. Additionally, EDTA is considered a potential pollutant with a low level of biodegradability (Bucheli-Witschel and Egli, 2001). In contrast, the biodegradability of GLDA makes it a more environmentally friendly feed component alternative to reduce dietary Zn supply, indirectly reducing Zn output in broiler production systems (Kołodyńska, 2013).

## Conclusions

The results of this study indicate that GLDA significantly increased the nutritional dietary availability of supplemental Zn in a manner and magnitude similar to that of EDTA in diets high in phytate and without phytase. GLDA has a positive effect on the nutritional availability of Zn present in the dietary ingredients. This study demonstrates that GLDA may be used as an effective supplement to increase the Zn bioavailability and to reduce the use of supplemental Zn levels in broiler diets. The effect of GLDA on the availability of native Cu, Mn, and Fe requires further study using a deficiency model for these nutrients.

## Abbreviations

AICC: Akaike information criterion
CCC: concordance correlation coefficient
DWG: daily weight gain
EDTA: ethylenediaminetetraacetic acid
GLDA: l-glutamic acid, N,N-diacetic-acid
ICPMS: inductively coupled plasma mass spectrometry
RMSE: root mean squared error
